# Spontaneous Subconjunctival Abscess: A Case Series and Review of the Literature

**DOI:** 10.7759/cureus.111677

**Published:** 2026-06-28

**Authors:** Andrew J Adamek, Asim V Farooq

**Affiliations:** 1 Ophthalmology, University of Chicago Medicine, Chicago, USA

**Keywords:** case reports, infectious disease, pediatric, spontaneous, subconjunctival abscess

## Abstract

Subconjunctival abscesses are exceedingly rare in the absence of previous ocular trauma or surgery. Here, we report three such cases and review the current literature. Three male patients (ages 3, 36, and 66 years) developed a spontaneous subconjunctival abscess without prior ocular trauma or surgery. The pediatric patient developed symptoms after swimming in a pool, while the adult patients were healthcare workers; all were immunocompetent. Conjunctival cultures were negative. Two cases were treated medically, either with topical moxifloxacin or fortified vancomycin and tobramycin, and resolved without recurrence. In the third case, the abscess worsened despite topical moxifloxacin and oral trimethoprim-sulfamethoxazole and ultimately required surgical excision followed by fortified vancomycin and tobramycin. All three cases resolved without recurrence at one month. In summary, spontaneous subconjunctival abscesses can occur in the absence of prior ocular trauma or surgery. This series includes the first reported pediatric case of spontaneous subconjunctival abscess and demonstrates the utility of AS-OCT in characterizing abscess morphology. Broad-spectrum antibiotics should be considered empirically, even with negative cultures, and worsening cases may require surgical excision.

## Introduction

Subconjunctival abscesses are rare, given the multilayered defense mechanisms of the conjunctiva. The protective barriers include (1) physical barriers consisting of epithelial tight junctions and mucin-producing cells [1,2], (2) antimicrobial peptides, including defensins, cathelicidins, and ribonuclease-7 [3,4], (3) conjunctiva-associated lymphoid tissue (CALT) [5], and (4) a highly vascular surface facilitating rapid immune cell recruitment [6]. Unless these protective barriers have been compromised, infection typically requires specialized virulence factors. For example, organisms such as nontypeable *Haemophilus influenzae* contain high-molecular-weight adhesion proteins (HMW1 and HMW2) that facilitate binding to conjunctival epithelial cells and IgA proteases, allowing potential invasion without prior tissue disruption [7-9]. Other organisms with a known capacity to invade intact ocular epithelium include *Neisseria gonorrhoeae*, *Neisseria meningitidis*, *Corynebacterium diphtheriae*, *Listeria monocytogenes*, and *Shigella* spp. [10], and *Acanthamoeba* spp. [11].

Therefore, subconjunctival abscesses are typically preceded by prior ocular trauma or surgery [12]. Reported cases of spontaneous development, without prior ocular surgery or trauma, are extremely rare (Table 1) [13-16]. Because of this rarity, risk factors have not been adequately characterized, and diagnosis remains a challenge, as early presentations may masquerade as severe conjunctivitis, episcleritis, or a retained foreign body. However, early recognition of this entity is critical; like other ocular infections, if untreated, a subconjunctival abscess may extend to adjacent ocular tissues and has the potential to cause infectious scleritis, keratitis, or endophthalmitis, all of which are vision-threatening complications [17]. Here, we report three cases of patients who developed spontaneous subconjunctival abscesses, including the first reported pediatric case without an underlying systemic condition. We also review the existing literature, which demonstrates the importance of recognizing this clinical entity.

## Case presentation

Case 1

A three-year-old previously healthy male presented with six days of redness in the outer corner of both eyes that developed the day after swimming in a pool. His mother reported that he developed mild crusting and mucus in both eyes for a few days, which resolved prior to presentation. The patient was also reported to have been irritable and to have had a fever for one day prior to presentation, which had also resolved. His pediatrician prescribed ofloxacin 0.3% TID OU. He denied pain or change in vision. There was no reported prior antibiotic use, signs, or history of immunocompromise, and the family denied any sick contacts.

On examination, the patient had visual acuities of 20/20 bilaterally. Slit-lamp examination revealed 2+ temporal injection with a subconjunctival abscess and a small overlying epithelial defect OD, and a temporal subconjunctival hemorrhage OS. Conjunctival cultures were obtained (all cultures in the series were obtained by swab, cultured for bacteria, and incubated for three days). The patient's medication was switched to moxifloxacin 0.5% drops every two hours while awake OD. At the two-day follow-up, the injection had improved, and the abscess had resolved with healed overlying epithelium. The conjunctival cultures were negative; because the systemic signs and symptoms had resolved prior to presentation, blood cultures were not obtained. At 10-day follow-up, the patient's ocular signs and symptoms had completely resolved, marked by the absence of the abscess and conjunctival injection on slit-lamp examination.

Case 2

A 36-year-old male physician who worked in an outpatient setting, with no significant ocular history, a systemic history notable only for parapsoriasis, no immunocompromise, and no recent prior antibiotic use, presented with redness and discharge OS for six days. He denied any pain, photophobia, or systemic symptoms. He had started using ciprofloxacin 0.3% QID OS without relief.

At his initial visit, visual acuities were 20/20 OD and 20/25 OS. Slit-lamp examination was normal OD. Slit-lamp examination revealed mild eyelid edema, trace chemosis, and 3+ bulbar conjunctival injection with mild discharge OS. Dilated fundus examination was within normal limits bilaterally. Cultures were obtained from the left inferior fornix, and he was initially treated with topical moxifloxacin 0.5% every two hours (Q2H) while awake.

At the one-week follow-up, a subconjunctival abscess had developed temporally OS. Cultures were obtained from the area of the abscess, and fortified vancomycin 20 mg/mL and tobramycin 15 mg/mL drops were started Q2H while awake. He was followed closely, and complete resolution of the abscess occurred within one week. Both sets of cultures were negative. No recurrence of the abscess was noted at one month.

Case 3

A 66-year-old male presented with redness of the right eye (OD) greater than the left eye (OS) for the last several days. He was a laboratory technologist who processed urine and blood samples and had a history of open-angle glaucoma treated with latanoprost and brimonidine/timolol. He had a history of atopic disease and herpes zoster dermatitis in the past year, but no immunocompromise or recent antibiotic use. He denied any pain or systemic symptoms.

At his initial visit, his visual acuities were 20/30 OD and 20/20 OS. Slit-lamp examination revealed palpebral and bulbar conjunctival follicles bilaterally and bulbar conjunctival injection OD greater than OS. Dilated fundus examination was deferred because of a recent examination with his glaucoma specialist. He was initially suspected to have either preservative toxicity or adult inclusion conjunctivitis. Conjunctival cultures were obtained from both eyes. Treatment was initiated with a single dose of azithromycin 1 g PO and prednisolone acetate 1% BID OU; additionally, his prostaglandin analog drop was changed to a benzalkonium chloride (BAK)-alternative formulation.

**Figure 1:**
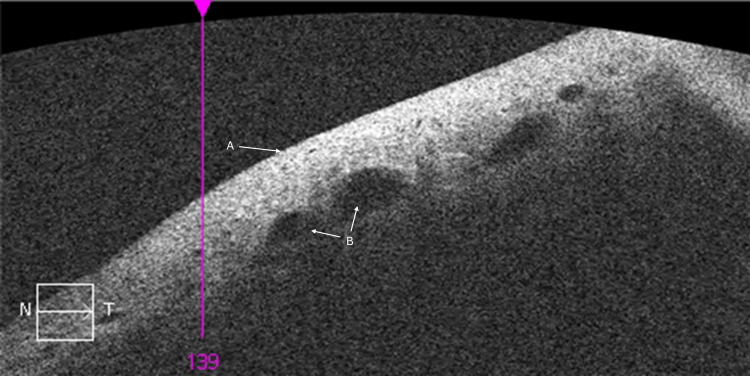
An anterior segment optical coherence tomography (AS-OCT) scan of case 3 revealing a multiloculated abscess temporally in the subconjunctival space. (A) Epithelial surface. (B) Multiloculated abscess within the subconjunctival space.

**Figure 2:**
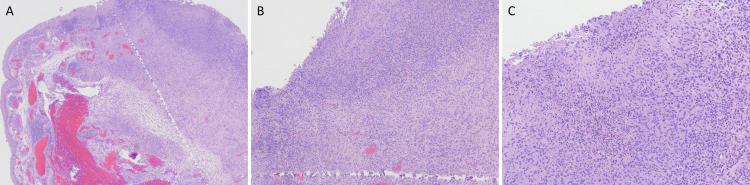
H&E stain of excised sample (case 3) at 4x (A), 10x (B), 20x (C) revealing a dense inflammatory cell infiltrate in the subconjunctival space without organisms with appropriate staining.

At the one-week follow-up visit, the follicular conjunctivitis had nearly resolved. The conjunctival cultures were negative. However, he was noted to have a subconjunctival abscess temporally OD. Topical moxifloxacin 0.5% drops Q2H while awake OD and oral trimethoprim-sulfamethoxazole (80 mg/400 mg) PO BID were prescribed and tolerated well. Three days later, the abscess was noted to worsen. High-definition anterior segment optical coherence tomography (AS-OCT) was performed for further evaluation and possible surgical planning. This imaging revealed a multiloculated abscess temporally in the subconjunctival space (Figure 1). After a discussion of the risks, benefits, and alternatives, surgical excision was performed. His treatment regimen was modified to fortified vancomycin 20 mg/mL and tobramycin 15 mg/mL drops, initially administered six times per day OD. No postsurgical recurrence of the abscess was noted at one month. Histopathology revealed a dense inflammatory cell infiltrate in the subconjunctival space without detectable organisms (Figure 2). Culture of the surgical specimen was negative for growth. Blood cultures were not obtained, given the absence of systemic signs and symptoms. 

## Discussion

Subconjunctival abscess formation is uncommon, with the vast majority of reported cases occurring in the setting of ocular surgery or trauma. This condition has been reported following strabismus surgery [12], scleral buckle surgery [18], glaucoma valve implantation [19], and posterior sub-Tenon triamcinolone acetonide injection [20]. The development of a subconjunctival abscess in the absence of ocular trauma or surgery suggests the role of an organism that is able to penetrate intact epithelium, such as Haemophilus influenzae, though *Staphylococcus *spp. have been reported, and in other reports, cultures have been negative [13-16]. One case was reported by Brooks et al. in 2010 [13], describing the development of an abscess near the left lateral canthus in a 27-year-old female that was draining at presentation. Cultures were positive for *Haemophilus influenzae*. She was treated initially with oral and topical moxifloxacin, as well as fortified vancomycin and tobramycin. Resolution was noted over several days. Mas-Castells et al. reported a spontaneous subconjunctival abscess in an adult patient. The patient was initially treated with chloramphenicol 0.5% eye drops for two weeks without improvement. Cultures grew *H. influenzae*, and resolution was achieved with one week of oral amoxicillin-clavulanate 500-125 mg three times daily, oral metronidazole 200 mg three times daily, and chloramphenicol 1% cream four times daily [14].

Some cases of subconjunctival abscess have also been documented in patients with underlying systemic conditions. Bubanale et al. reported a case of an infant with congenital lamellar ichthyosis who developed a subconjunctival abscess draining at presentation, with grade 3 upper eyelid ectropion and thickening of the anterior lamella. Resolution was achieved within two weeks with a course of intravenous ceftriaxone, amikacin, carboxymethylcellulose, and moxifloxacin 0.5% eye drops six times per day. Cultures from the abscess were positive for methicillin-resistant *Staphylococcus aureus *[15]. Additionally, Kukimoto et al. reported an HLA-B51-positive patient with ulcerative colitis (UC) who was treated for eye redness with a steroid eye drop. Ten days later, the patient was admitted with acute panuveitis and presented with a subconjunctival abscess in the inferonasal area, along with pustular rashes on the abdomen and face, a pansystolic murmur, and a fever. Cultures from the abscess were negative. Neutrophil infiltration into the dermis and subcutaneous adipose tissue was noted, and a diagnosis of aseptic abscess associated with UC was made. The patient was treated with high-dose glucocorticoid therapy along with infliximab infusion, resulting in complete resolution of the subconjunctival abscess [16].

In comparison to prior published cases, we present unique demographics. Cases 2 and 3 were healthcare workers; while neither patient reported specific pathogen exposures to the eye, general occupational exposure to pathogens in clinical settings may warrant consideration. Furthermore, Cases 2 and 3 also had underlying dermatologic conditions: parapsoriasis in Case 2 and atopic dermatitis in Case 3. Notably, the Bubanale et al. case demonstrated potential direct involvement of a dermatologic condition in the development of a subconjunctival abscess [15]; psoriasis and atopic dermatitis have been linked to an increased risk of conjunctivitis and ophthalmic infection, potentially via an aberrant immune response and/or epithelial barrier dysfunction [21,22]. Finally, to our knowledge, we also present the first case of a spontaneous subconjunctival abscess in an otherwise healthy pediatric patient.

Regarding the diagnostic approach, our cases had negative cultures, which added diagnostic complexity and required empiric treatment. It is possible that, since our cases were not draining spontaneously and the abscesses appeared to be well encapsulated, a positive yield with swab cultures was more difficult to achieve. Case 2 was also being treated with topical antibiotics prior to the first culture, which may have limited culture growth. Additionally, we only sent bacterial cultures; the standard incubation time for bacterial ophthalmic cultures at our institution's laboratory is three days. These factors inherently limit the yield for slower-growing bacteria such as *Nocardia *spp., atypical mycobacteria, *Acanthamoeba*, as well as fungi [23]. Furthermore, we cannot exclude sterile inflammatory collections, as suggested by the sterile abscess in the setting of ulcerative colitis in the Kukimoto et al. case [16], especially given the underlying inflammatory conditions in Cases 2 and 3. However, the rapid response to broad-spectrum antibiotics in these cases supports, although does not necessarily confirm, an infectious etiology. We acknowledge the limitation of the culture-negative status in this series and highlight these findings to support consideration of longer incubation times, as well as fungal cultures when appropriate. We also acknowledge our small sample size, short follow-up, and the need for further reported cases to guide evidence-based management.

## Conclusions

Clinicians should be aware that spontaneous subconjunctival abscesses may occur in the absence of previous ocular trauma or surgery. Based on our cases, we offer several clinical considerations. Although cultures may be negative, broad-spectrum antibiotics were effective in the resolution of Cases 1 and 2. In Case 3, which required procedural intervention, AS-OCT proved valuable in characterizing the multiloculated nature of the abscess and allowing noninvasive visualization of the subconjunctival collection, helping guide surgical planning. In our experience, surgical excision was effective for the definitive management of a worsening, refractory abscess, though further cases are needed to establish surgical indications.
